# Study of Sodium Ion Selective Electrodes and Differential Structures with Anodized Indium Tin Oxide

**DOI:** 10.3390/s100301798

**Published:** 2010-03-04

**Authors:** Jyh-Ling Lin, Hsiang-Yi Hsu

**Affiliations:** Department of Electronic Engineering, HuaFan University, No.1, Huafan Rd., Shihting Hsiang, Taipei Hsien, 223 Taiwan; E-Mail: m9626024@cat.hfu.edu.tw

**Keywords:** anodized indium tin oxide, anodic oxidation, sodium ion, EGFET

## Abstract

The objective of this work is the study and characterization of anodized indium tin oxide (anodized-ITO) as a sodium ion selective electrode and differential structures including a sodium-selective-membrane/anodized-ITO as sensor 1, an anodized-ITO membrane as the contrast sensor 2, and an ITO as the reference electrode. Anodized-ITO was fabricated by anodic oxidation at room temperature, a low cost and simple manufacture process that makes it easy to control the variation in film resistance. The anodized-ITO based on EGFET structure has good linear pH sensitivity, approximately 54.44 mV/pH from pH 2 to pH 12. The proposed sodium electrodes prepared by PVC-COOH, DOS embedding colloid, and complex Na-TFBD and ionophore B12C4, show good sensitivity at 52.48 mV/decade for 10^−4^ M to 1 M, and 29.96 mV/decade for 10^−7^ M to 10^−4^ M. The sodium sensitivity of the differential sodium-sensing device is 58.65 mV/decade between 10^−4^ M and 1 M, with a corresponding linearity of 0.998; and 19.17 mV/decade between 10^−5^ M and 10^−4^ M.

## 1. Introduction

Sodium detection in blood, food, and soil is increasingly important. The sodium ion is one of the essential mineral substance nutrients in the human body. *In vivo*, sodium mostly exists in the blood and the extracellular fluid, where with potassium it maintains the balance of osmotic pressures and moisture inside and outside the cell and assists in the normal operation of nerves, heart, muscles, and other physiological functions. However, people may consume excessive sodium easily causing hypertension. Therefore, strictly controlling sodium content in food is very important for modern people, in particular for fast food addicts.

Determination of sodium content in the blood is important to specific clinical and medical applications because of the mentioned participation in the human body’s metabolic processes. Sodium content is related to heart disease, making it an important factor in the study of hypertension, hypopotassemia, alkalosis, cirrhosis of the liver, and diuretic drugs [1,2]. Hence, a method that is accurate, easy, and quickly generates test results for levels of the sodium ion will have useful applications.

In the human body, normal sodium concentration is from 0.135 M to 0.145 M. In this study, high performance sensors, based on extended-gate ion-sensitive field effect transistors (EGFET), were realized. The sensor’s sensitivity to sodium ions is 52.48 mV/decade for a concentration between 0.01 M and 1 M using the potentiometry measurement. Compared with conventional electrodes, this ion-selective electrode based on potentiometric detection is a simple method, and offers several advantages such as rapid and easy use of simple procedural instruments, speed and ease of preparation, relatively fast response, wide dynamic range, reasonable selectivity, low cost, and developing to a portable-like, automation, and immediate examination product. In addition, they are ideally suited for wide use in diverse clinical and medical fields [3].

Indium tin oxide/glass (ITO/glass) is widely used as the substrate conductive layer in pH sensors or biosensors [4,5]. This study aimed to examine the possibility of using it as the detection membrane because it contains tin oxide which is a good acid-base detective membrane. However, the resistance of commercial ITO, with several sheets of resistance used in optoelectronics as the transparent electrode, is too low to be a pH sensing membrane. So, anodic oxidation was used to treat ITO, forming a high resistance ITO membrane on ITO/glass which could own high acid-base sensitivity and stability.

## 2. Experimental

### 2.1. Experimental Methods for Preparing Anodized Indium Tin Oxide Membrane

Anodized indium tin oxide was fabricated from indium tin oxide/glass by anodic oxidation. First, the ITO/glass is cleaned to remove the oil and then dried in an oven. Then the ITO/glass is patterned by lithography and etched as a multi-window; the window area is fixed at 2 × 2 mm^2^. Finally, the patterned ITO is treated by anodic oxidation. An electrolyte consisting of tartaric acid, ethyl alcohol, and high-purity deionized water (DI water) in the ratio of 3.5 g: 250 mL: 50 mL is used in this study. The developed films have a fine surface, grow slowly, and are easily controlled [6].

**Figure 1.**
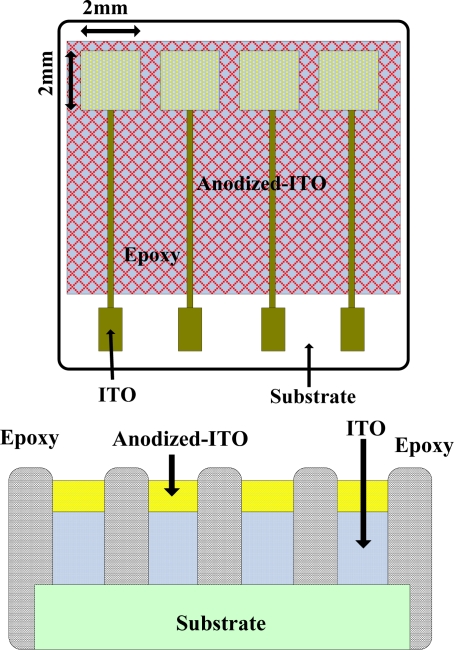
Planar view and cross section of anodized-ITO pH electrode.

A fixed current density is adopted in anodic oxidation; the optimal current condition herein is 0.6 mA/cm^2^. The resistance of indium tin oxide is maintained at 400 kΩ to maximize the sensitivity of the sensor to the acid-base. The calculated resistance of anodic indium tin oxide is:

(1) $$R_{\mathrm{AITO}}=\frac{V_{\mathrm{End}}-V_{\mathrm{Start}}}{I}$$

where R_AITO_ is the resistance of anodic indium tin oxide, V_End_ is the final voltage of the voltmeter, V_Start_ is the initial voltage of the voltmeter, and I is the fixed current. The top view and cross-section of the 1 × 4 multi-window anodized-ITO/ITO/glass are shown in Figure 1.

**Figure 2.**
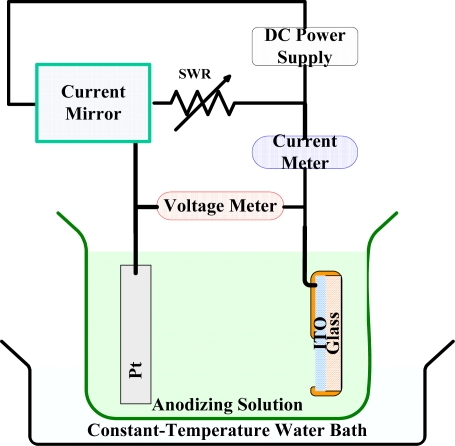
An indium tin oxide anodic oxidation system. The current mirror produced a constant current, and the optimal current condition is 0.6 mA/cm^2^. An electrolyte consisting of tartaric acid, ethyl alcohol, and DI water in the ratio of 3.5 g: 250 cc: 50 cc is used.

Figure 2 shows the anodic oxidation system. The current mirror produced a constant current, and the optimal condition for indium tin oxide is 0.6 mA/cm^2^.

### 2.2. Materials

The following components were used for the sodium membrane preparation: poly (vinyl chloride) carboxylated (PVC-COOH), and bis(2-ethylhexyl) sebacate (DOS) were purchased from Sigma; sodium ionophore VI (B12C4), sodium tetrakis(4-fluorophenyl)borate dihydrate (Na-TFBD) were purchased from Fluka; sodium chloride and tetrahydrofuran (THF) used as a solvent were purchased from J. T. Baker; standard solutions were prepared with deionized water and all the other reagents used in this study were all purchased through Uni-onward Corp. in Taiwan. The buffer solution for the sodium standards was blended by EDTA 1.0 mmol/L and Tris 20 mmol/L with 1 L of DI water. Then the solution was added to 116.88 g of sodium chloride. Finally, the solution was adjusted to pH 7.5 with 0.5 M HCl for the optimum measuring conditions.

### 2.3. Preparation of Sodium Ion Selective Electrodes

Preparation of sodium ion selective electrodes was such that 33 mg of the main PVC-COOH was dissolved in 0.375 mL of tetrahydrofuran and the solution plasticizer (DOS), ionophore (B12C4), and lipophilic salt (Na-TFBD) were added to it. The final membrane composition was formed by the main materials including PVC-COOH (33 mg) and the plasticizer DOS (66 mg). Then, the sodium selective sensor used ionophore B12C4 and Na-TFBD as the selector. The mixture was thoroughly stirred and 2 mL of ion-selective compound was absorbed onto the window. Finally, the ion selective sensors were stored at room temperature for 12 to 24 h [7–9].

### 2.4. Fabrication of Differential Sodium Ion Selective Electrodes

**Figure 3.**
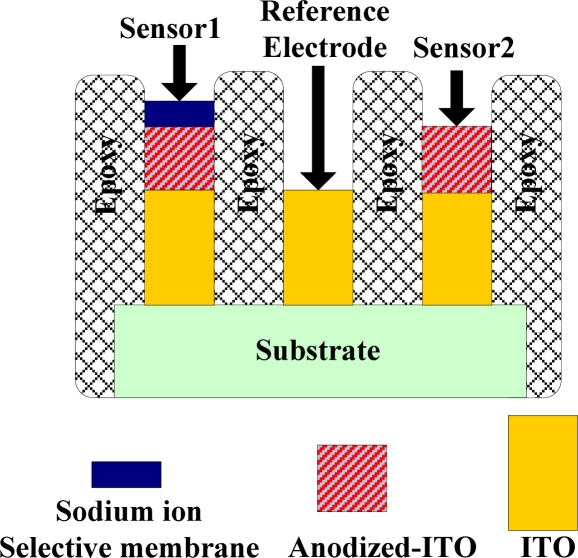
Cross sectional view of the differential sodium-sensing device. Sensor 1 is sodium-selective-membrane/anodized-ITO/ITO, sensor 2 is anodized-ITO/ITO, and the reference electrode is ITO.

A differential sodium ion selective electrode, based on ITO, includes three electrodes, an ion-selective-membrane/anodized-ITO as the sensor 1, an anodized-ITO membrane as the contrast sensor 2, and an ITO as the reference electrode. The ITO electrodes were designed to manufacture a miniaturized sensor on ITO substrate by photolithography and chemical etching. Then the ITO windows of sensor 1 and sensor 2 were treated simultaneously by anodic oxidation with 400 kΩ resistance. The other ITO/glass window was used as the reference electrode because the ITO is the conductive material with 10 sheet resistance, so it can serve as the electrical contact to define the electrical potential of the electrolyte solution. Finally, a sodium-selective compound was embedded on the sensor 1 anodized-ITO membrane. This structure is simple, convenient to manufacture, and the total area is smaller than 1 cm^2^, because the detection window area of each is designed at 2 × 2 mm^2^, which are all shown in Figure 3.

### 2.5. Measurement System

**Figure 4.**
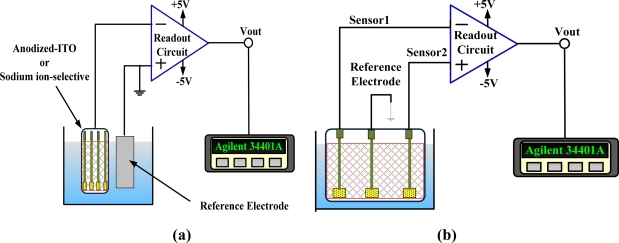
Measurement structures of (a) pH and single sodium electrode, and (b) differential sodium sensor.

In order to measure the potential of the anodized-ITO/ITO and sodium-selective-membrane/anodized-ITO/ITO in the corresponding solution, the instrument pre-signal circuit is used as the readout circuit. It’s a very effective circuit for use in potentiometric biochemical sensors. Figure 4(a) shows the schematic of the measurement system which is used to obtain the pH sensitivity and sodium sensitivity for the anodized-ITO/ITO and sodium-selective/anodized-ITO/ITO. The other measurement structure, to measure the potential of the differential sodium-sensing device, is shown in Figure 4(b).

## 3. Results and Discussion

### 3.1. Anodized Indium Tin Oxide Characteristics

The color of anodized-ITO is clearly changed because of the formation of tin oxide. Auger Electron Spectroscopy (AES) was used to study the surface composition and thickness of anodized-ITO. The thickness of anodized-ITO is about 40 Å. The composition ratios of ITO and anodized-ITO are In:Sn:O = 37:3:60 and In:Sn:O = 31.45:2.55:66.0, showing the increase of oxygen after anodic oxidation treatment.

**Figure 5.**
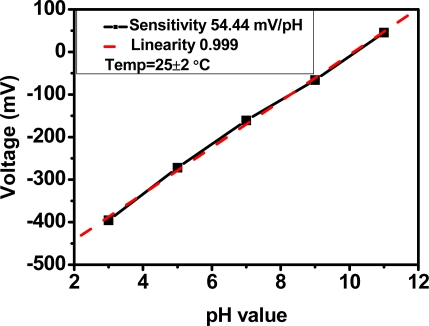
pH sensitivity of the anodized-ITO/ITO electrode.

The prepared anodic indium tin oxide, which connects the commercial reference electrode with the instrumentation amplifier forming an EGFET structure, was placed in acidic and basic solutions to determine acid-base characteristics. Figure 5 shows the output voltage *versus* time for different pH values. The sensitivity is 54.44 mV/pH and the linearity is 0.999. It is verified that anodic indium tin oxide has acid-base sensitivity and a high reproducibility when ITO/glass is treated by anodic oxidation.

### 3.2. Results of Sodium Ion Selective Electrodes

**Figure 6.**
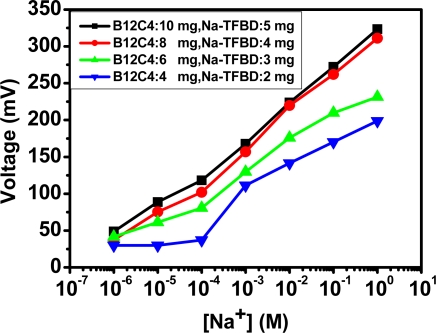
Sodium-sensing sensitivity of sodium-selective-membrane/anodized-ITO/ITO with four ratios of selective membrane.

The following discussion concerns the characteristics of the sodium ion selective electrode. All measurements used the structure shown in Figure 4(a) and 50 mL buffer solution as the standard. The detection solutions are obtained by titration and fixed at about 50 mL. The electrodes were calibrated when the linearity exceed 0.99 in 10^−4^, 10^−2^, and 1 M solution. First, Figure 6 shows the influence of sodium detection with various selective membranes on anodized-ITO. This study uses a different count of complex Na-TFBD and ionophore B12C4, trying to use the least count to obtain the optimum condition. The results display 10 mg B12C4, 5 mg Na-TFBD and 8 mg B12C4, with a 4 mg Na-TFBD good sodium sensitivity and they are not very different. Therefore, the last formula for our standard process is selected.

**Figure 7.**
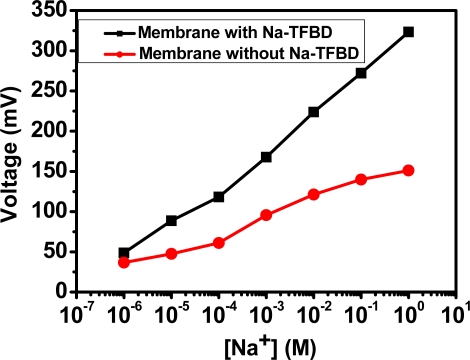
Sodium-sensing sensitivity of sodium-selective-membrane/anodized-ITO/ITO with Na-TFBD and without Na-TFBD.

The influence of adding Na-TFBD was also studied in this paper. Figure 7 shows the sodium detection membranes with and without Na-TFBD complex. The sensitivity of ‘with complex’ is better than that of ‘without complex’ because the complex anion of Na-TFBD can be dispersed in the compound between the solution and the high polymer monomer, increasing the electro-negativity of a macromolecule to attract the positive-charge ions and raising its selective potential.

**Figure 8.**
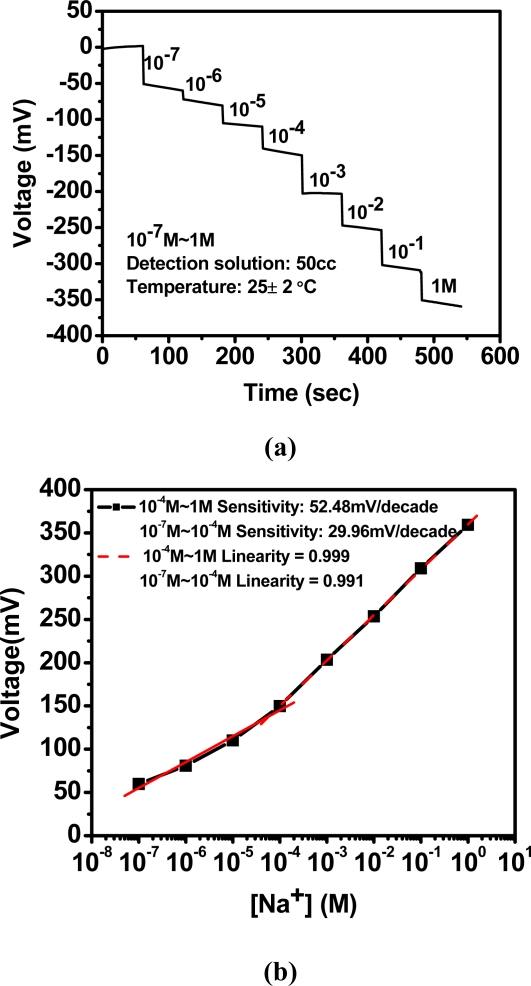
Sodium ion selective electrode based on anodized-ITO for (a) output response voltage *vs*. times with titration and (b) corresponding sensitivity.

Figure 8(a) illustrates the relationship between concentrations and voltage response to the sodium ions detection by titration. Using the titration method is to consider fixing the measurement condition, to avoid disturbance from temperature, solution *etc.* Figure 8(b) shows the corresponding sodium sensitivity which is 52.48 mV/decade for 10^−4^ M to 1 M, and 29.96 mV/decade for 10^−7^ M to 10^−4^ M.

In this paper, the disturbance from different ions was studied using the Separate Solution Method (SSM) [10–12]. The concentrations of a cell comprising an ion-selective electrode and a reference electrode (ISE cell) are adjusted with each of the two separated-solutions, one containing the primary ion, A, with the concentration a_A_ (but no B), the other containing the interfering ion, B with the concentration a_B_ as high as required to achieve the same measured cell voltage (E_A_ = E_B_). From any pair of concentrations a_A_ and a_B_ giving the same cell voltage, the value of potentiometric selectivity coefficient may be calculated from the equation (2):

(2) $$K_{A,B}^{\mathrm{pot}}=\frac{a_{A}}{{a_{B}}^{z_{A}/z_{B}}}$$

where Z_A_ is the charge number, an integer with sign and magnitude corresponding to the charge of the primary ion, A; Z_B_ is the charge number corresponding to the charge of interfering ion, B.

**Figure 9.**
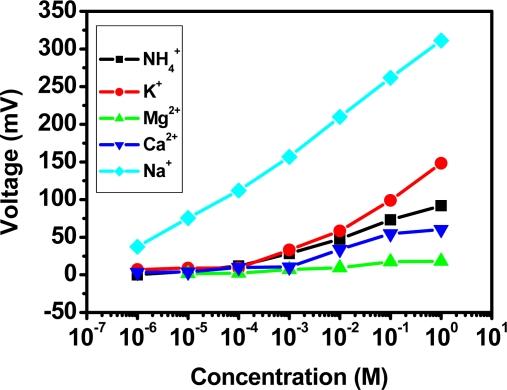
Interference study by Separate Solution Method for sodium ion selective electrode.

Figure 9 shows four reactions based on a sodium ion selective electrode in NH_4_^+^, K^+^, Ca^+2^, and Mg^+2^ perturbed ion solutions, respectively. The corresponding $K_{A,B}^{\mathrm{pot}}$ values are summarized in Table 1. There is almost no serious interference except for the concentrations over 10^−3^ M for K^+^-ions and NH_4_^+^-ions.

**Figure 10.**
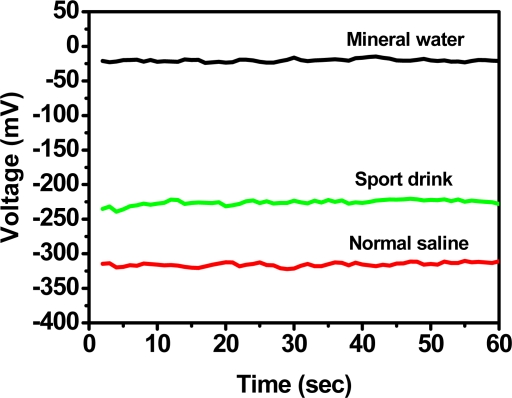
Sodium ions test in commercial mineral water, sport drinks, and physiological salt water with sodium-selective-membrane/anodized-ITO/ITO electrode.

To check the possible application of anodized-ITO sodium ion selective electrodes, detection solutions were used from commercial mineral water, sport drinks, and physiological salt water. Figure 10 displays the results of these three solutions. The indicated-content of sodium in the sport drinks is 7.7 mM and the physiological salt water is 154 mM.

### 3.3. Results of Differential Sodium Ion Selective Electrodes

**Figure 11.**
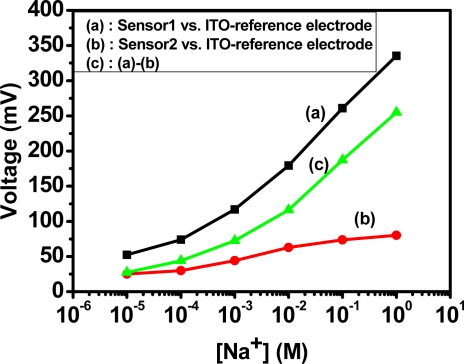
Sodium sensitivities of (a) the sodium-selective-membrane/anodized-ITO/ITO structure, (b) the anodized-ITO/ITO structure, and (c) the calculation of (a)–(b).

The foregoing discussion focused on studying the characteristics of anodic indium tin oxide sodium ion-selective electrodes. Following is a differential structure based on indium tin oxide, and substitution of the reference electrode will be investigated. For the differential ISFET, there are two detectors and one reference electrode in the system. In this study, ITO/glass is used as the reference electrode. Sensors 1 and 2, which use the ITO reference electrode to identify the potential between these two sensors, have different sodium sensitivities. The output voltage of this differential system can be expressed as follows:

(3) $$V_{\mathrm{out}}=V_{1}-V_{2}=(V_{\mathrm{sen}1}-V_{\mathrm{ref}})-(V_{\mathrm{sen}2}-V_{\mathrm{ref}})=V_{\mathrm{sen}1}-V_{\mathrm{sen}2}$$

where *V_ref_* is the potential of the reference electrode that is connected to ground in our system, *V*_*sen*1_ is the potential of sensor 1, *V*_*sen*2_ is the potential of sensor 2, *V*_1_ is the various potentials between sensor 1 and the reference electrode, and *V*_2_ is the various potentials between sensor 2 and the reference electrode. The reference electrode only provides the base potential for these two sensors, so conductor material, such as Pt and low resistance ITO, was used in this study. Considering simple structure and process, the same base substrate, and cheap ITO were used. The responses of an anodized-ITO/ITO structure and a sodium-selective-membrane/anodized- ITO/ITO structure *versus* an ITO reference electrode were calibrated by the schematic of a measurement system, which is shown in Figure 4(a). The results are shown in Figure 11 curves (a) and (b).

**Figure 12.**
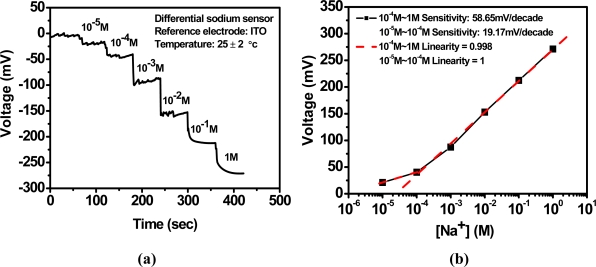
Differential sodium ion sensor based on anodized-ITO for (a) output response voltage *vs.* times with titration and (b) corresponding sensitivity.

Curve (b) shows low sodium response because sensor 2 isn’t embedded in an ion-selective membrane. Curve (c) is the result of (a) minus (b). The output response of the differential sodium sensor used Figure 4(b)’s differential measurement system is shown in Figure 12(a). The sensitivity is shown in Figure 12(b), which is 58.65 mV/decade between 10^−4^ M and 1 M, with the corresponding linearity being 0.998; and 19.17 mV/decade between 10^−5^ M and 10^−4^ M, which is better than Figure 11 curve (c) because the differential structures cancel the noise from sensor 1 and sensor 2. This differential sodium sensor has high linear sensitivity and good reliability and reproducibility. In addition this structure is suitable to be developed as a sodium sensor, array structures, and multi-sensors because of its simple fabrication, low cost, good characteristics, and high integration.

## 4. Conclusions

Ion-selective sensors fabricated by anodic oxidation on ITO substrates were used in the EGFET structure. Anodic oxidation technology is simple, low cost, and can be used for mass production. A sodium-selective sensor and differential sodium-selective sensor on anodized-ITO were analyzed in this paper, and the structure is suitable to be developed as an array structures and multi-sensors because of its simple fabrication, low cost, good characteristics, and high integration.

The conclusions may be summarized as follows:

1. Indium tin oxide treated by anodic oxidation at room temperature is used as an EGFET sensing film. The resistance of the anodized-ITO is fixed at 400 kΩ, its pH sensitivity is 54.44 mV/pH, and the linearity is 0.999.
2. The proposed sodium electrodes were prepared using PVC-COOH, DOS embedding colloid, and complex Na-TFBD and ionophore B12C4 on an anodized-ITO on commercial ITO coated glass. Measurement of sodium ions showed a good sensitivity at 52.48 mV/decade for 10^−4^ M to 1 M and 29.96 mV/decade for 10^−7^M to 10^−4^ M. In the SSM (E_A_ = E_B_) interference measurement, there is almost no serious interference except for concentrations over 10^−3^ M for K^+^-ions and NH_4_^+^-ions.
3. The differential sodium sensor uses ITO/glass as electrical contacts and reference electrodes because ITO is the conductive material. The anodized-ITO/ITO has lower sodium sensitivity than the sodium-selective-membrane/anodized-ITO/ITO. The sodium sensitivity of the differential sodium-sensing device is 58.65 mV/decade between 10^−4^ M and 1M, with a corresponding linearity of 0.998; and 19.17 mV/decade between 10^−5^ M and 10^−4^ M.

## Figures and Tables

**Table 1.** Selectivity coefficients of Na^+^- selective electrode by Separate Solution Method (E_A_ = E_B_).

	${\mathrm{NH}}_{4}^{+}$	***K^+^***	***Ca^+2^***	***Mg^+2^***
$\log\;K_{{A,B}^{+}}^{\mathrm{pot}}$	**−4.06**	**−3.11**	**−5.65**	**−6.76**

## References

[1] Komaba S., Arakawa J., Seyama M., Osaka T., Satoh I., Nakamura S. (1998). Flow injection analysis of potassium using an all-solid-state potassium-selective electrode as a detector. Talanta.

[2] Ermolenko Y., Yoshinobu T., Mourzine Y., Furuichi K., Levichev S., Schoning M., Vlasov Y., Iwasaki H. (2003). The double K^+^/Ca^2+^ sensor based on laser scanned silicon transducer (LSST) for multi-component analysis. Talanta.

[3] Abramova N., Borisov Y., Bratov A., Gavrilenko P., Domínguez C., Spiridonov V., Suglobova E. (2000). Application of an ion-selective field effect transistor with a photocured polymer membrane in nephrology for determination of potassium ions in dialysis solutions and in blood plasma. Talanta.

[4] Pan C.W., Chou J.C., Kao I.K., Sun T.P., Hsiung S.K. (2003). Using polypyrrole as the contrast pH detector to fabricate a whole solid-state pH sensing device. IEEE Sens. J.

[5] Hsu H.Y., Wu C.Y., Lee H.C., Lin J.L., Chin Y.L., Sun T.P. (2009). Sodium and potassium sensors base on separated extended gate field effect transistors. Biomed. Eng. Appl. Basis Commun.

[6] Lin J.L., Chu Y.M., Hsaio S.H., Chin Y.L., Sun T.P. (2006). Structure of anodized aluminum oxide extended-gate field-effect transistors on pH sensors. Jpn. J. Appl. Phys.

[7] Lee Y.H., Elizabeth A.H. (2001). Hall assessing a photocured self-plasticised acrylic membrane recipe for Na+ and K+ ion selective electrodes. Anal. Chim. Acta.

[8] Chandra S., Lang H. (2006). A new sodium ion selective electrode based on a novel silacrown ether. Sens. Actuat. B.

[9] Chou J.C., Huang Y.P. (2008). Fabrication and stability analysis for sodium ion sensor. Sens. Lett.

[10] Marques de Oliveira I.A., Risco D., Vocanson F., Crespo E., Teixidor F., Zine N., Bausells J., Samitier J., Errachid A. (2008). Sodium ion sensitive microelectrode based on a p-tert-butylcalix[4]arene ethyl ester. Sens. Actuat. B.

[11] Umezawa Y., Buhlmann P., Umezawa K., Tohda K., Amemiya S. (2000). Potentiometric selectivity coefficients of ion-selective electrodes Part I. Inogranic cations. Pure Appl. Chem.

[12] Lindner E., Umezawa Y. (2008). Performance evaluation criteria for preparation and measurement of macro- and microfabricated ion-selective electrodes. Pure Appl. Chem.

